# Vital sign-based Early Warning Scores in low- and middle-income countries: a systematic review of clinical effectiveness and implementation challenges

**DOI:** 10.1080/16549716.2026.2690321

**Published:** 2026-06-17

**Authors:** Niels Jig Jansen, Lars Ingmar Veldhuis

**Affiliations:** Department of Intensive Care, Saint Francis Hospital, Mutolere, Uganda

**Keywords:** Low resource setting, mortality, Prediction models, serious adverse events, vital signs

## Abstract

Early recognition of clinically deteriorating patients may improve patient outcomes. Early warning scores (EWS) are widely used in high-income settings, but their effectiveness in low- and middle-income countries (LMICs) remains uncertain. This systematic review evaluates whether the implementation of EWS improves patient outcomes in LMICs and explores factors that may influence their effectiveness. A systematic search was conducted in Pubmed, EMBASE, and the Cochrane Library from database inception to 12 December 2024. Studies comparing patient outcomes before and after EWS implementation in LMICs were included. Outcomes of interest were mortality, length of hospital stay, serious adverse events (SAEs), and intensive care unit (ICU) admissions. Five studies met the inclusion criteria. Overall, EWS implementation was not associated with a consistent reduction in mortality or SAEs. However, two studies reported a shorter length of hospital stays following implementation, and one study demonstrated a reduction in unplanned ICU admissions. The included studies were heterogeneous in design, setting, and patient populations and therefore overall certainty of evidence was low to moderate. Current evidence does not demonstrate an improvement in patient outcomes following the implementation of EWS in LMICs. However, this finding is based on a small number of heterogenous studies with varying methodological quality and should therefore be interpreted with caution. The absence of observed benefits may reflect both limitations of the available evidence and contextual factors, such as limited resources, insufficient training, and restricted escalation capacity. Future research should focus on both clinical effectiveness and implementation factors within LMIC health-care systems.

## Paper Context

- **Main findings:** In contradiction to high-income countries, this review does not demonstrate improved patient outcomes following EWS implementation in low- and middle-income countries.
- **Added knowledge:** EWS alone may be insufficient to improve patient outcomes in low resource settings. Inadequate health systems including limited health-care resources, insufficient training, and restricted escalation capacity may prevent effective implementation of EWS.
- **Global health impact for policy and action:** This review highlights the importance of holistic and standardized implementation of new health systems like EWS. If the circumstances are not optimized, new implementations alone will not reach their potential.

## Background

Early detection of clinical deterioration in hospitalised patients is essential to enable timely interventions that may prevent serious adverse events (SAEs) or death. To support in this process, early warning scores (EWS) have been developed to assist health-care professionals in systematically monitoring and assessing patient conditions. The EWS facilitate the early identification of physiological changes indicative of potential deterioration. The EWS are designed to identify a deteriorating patient up to 24 hr prior to the event, allowing for early interventions and the prevention of adverse outcomes [1–3]. The scores give a standardised classification of the degree of physiological abnormality based on the heart rate, blood pressure, respiration rate, temperature, conscious level, and peripheral oxygen saturation, with higher scores representing a higher risk of deterioration.

Previous studies have suggested that 17%–75% of SAEs may be preventable [4–6]. This highlights the importance of early intervention when an elevated EWS is detected. Many hospitals have established escalation protocols requiring nurses to alert physicians when scores reach critical levels. In the event of a delay or unavailability of the physician, the NICE guidelines recommend involving the ICU team directly to ensure timely escalation of care [7,8]. While a high EWS does not always necessitate immediate ICU admission, it signals the need for intensified monitoring or a potential transfer to higher levels of care. The overarching goal of EWS is to enable swift and appropriate responses that stabilize patients and prevent severe complications.

Since their introduction in 1997, EWSs have been widely adopted in various clinical settings, particularly in high-income countries, where they are now a standard component of clinical practice [2,9]. Despite their widespread use in high-income countries, the evidence supporting EWS remains limited, partly due to the considerable heterogeneity among scoring systems and study designs [5]. However, it remains uncertain whether EWS is beneficial in low-resource settings, where higher mortality rates are frequently associated with limited access to trained personnel, essential equipment, and structured clinical systems. Implementing EWS in these environments could represent a significant step toward improving patient outcomes, but challenges unique to these settings may impact their effectiveness.

This systematic review evaluates the impact of EWS in resource-limited health-care systems. By synthesizing evidence from studies conducted in these regions, this review aims to shed light on the opportunities and obstacles associated with implementing EWS in environments with constrained resources. The findings may provide valuable insights into how EWS could be adapted to improve care and reduce preventable adverse outcomes in these settings.

## Methods

### Selection criteria

Studies were eligible if they were randomized trials, comparative observational studies, or controlled before-and-after (CBA) studies. All included studies required a control group and could involve patients of any age group. The study must have been conducted in a low-and-middle-income country (LMIC), according to the World Bank‘s definition. The intervention of interest was the implementation of an EWS system, including the Modified Early Warning Scores (MEWS), Paediatric Early Warning Scores (PEWS), or National Early Warning Scores (NEWS). Outcome definitions of interest included mortality, ICU admission, SAE, and other patient-related outcomes.

Exclusion criteria included studies without a control group, missing data, or unavailable full text. Studies published in languages other than English were also excluded. This systematic review was conducted in accordance with Preferred Reporting Items for Systematic reviews and Meta-Analyses (PRISMA) guidelines.

### Search strategy

A comprehensive literature search was conducted in PubMed, EMBASE, and the Cochrane Library from database inception to 12 December 2024. The search strategy combined Medical Subject Headings (MeSH) and free-text terms related to EWS, patient outcomes, and low- and middle-income countries (LMICs). The full search strategy is provided in Supplementary Appendix 1. Search results were imported into Rayyan (Qatar Computing Research Institute), where duplicates were removed.

### Study selection

Two authors (NS and LV) independently reviewed all potentially relevant titles and abstracts to ensure consistency and minimise bias. Thereafter, full-text articles were assessed to confirm their eligibility based on the predefined inclusion criteria.

Disagreements during the selection process were resolved through discussions among the reviewers until a consensus was achieved.

### Data extraction and analysis

Data were manually extracted independently by the reviewers using a standardized Microsoft Excel data form. Key information collected included author, study design, country, setting, participant characteristics (e.g. number and age), interventions performed, comparators (e.g. control groups), and outcomes (e.g. mortality, ICU admission, and SAE). A narrative synthesis was performed to summarize the results.

### Risk of bias and quality assessment

The certainty of evidence was assessed using the GRADE (Grading of Recommendations Assessment, Development, and Evaluation) tool. The initial certainty of evidence was determined by the study design, with randomized trials rated as high certainty and observational studies rated as low certainty.

Certainty was downgraded based on factors, such as risk of bias, inconsistency, indirectness, imprecision, or publication bias. It could be upgraded to attributes like a large effect size. The final certainty levels for these outcomes were categorized as high, moderate, low, or very low, reflecting the cumulative impact of these factors.

## Results

### Study selection

**Figure 1.**
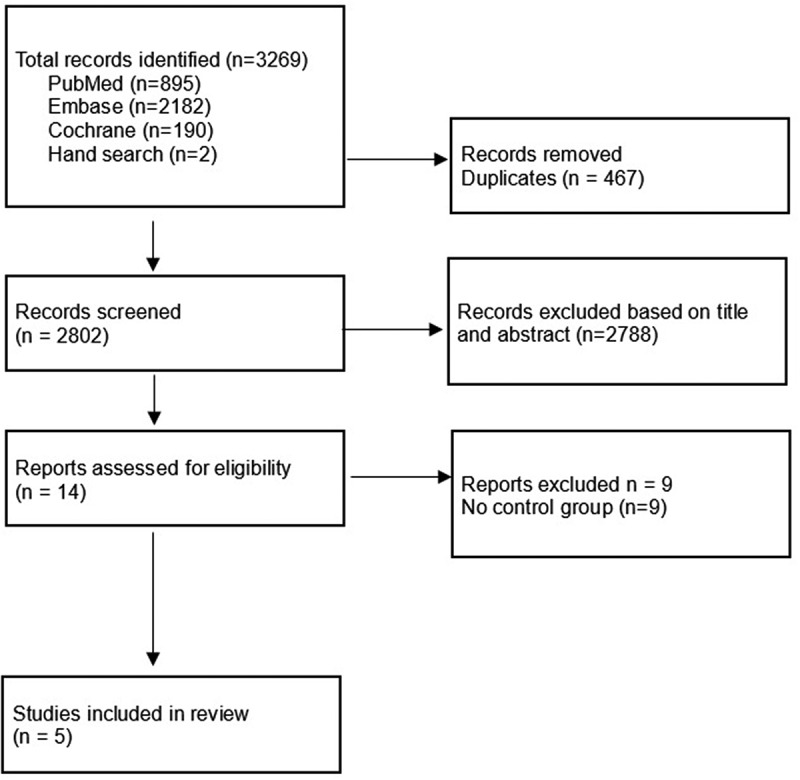
PRISMA flow diagram.

Through database searches, a total of 3,269 articles were identified of whom 467 duplicates were removed. A total of 2,802 articles were screened based on the title and abstract. A total of 14 articles were read and assessed for the eligibility criteria. A total of five studies met the inclusion criteria (Figure 1).

### Study characteristics

Two of the included studies [10,11] were multicentre studies. In these studies, the intervention and the control group were collected from different hospitals. The other three included studies [12–14] showed the implementation of an EWS in a single centre. The type of EWS used varied between studies. One of the studies [10] implemented the paper-based Cape Town (CT) MEWS. Two studies [12,13] used the PEWS, of which one study did some modification and simplification to make it more user-friendly for less trained staff. Another study [14] implemented the NEWS, and the last study [11] implemented the obstetric EWS. Type of studies were randomized control trails, before and after implementation or case control. All studies consisted of two groups, in which various factors were examined both before and after the implementation of the EWS. All the studies were performed in low-income countries. Study characteristics are summarised in Table 1.

**Table 1.** Overview of characteristics of the included articles.

Study	Setting	EWS	*N*= (control vs intervention)	Primary Outcome	Main Findings
Kyriacos et al. [10]	South Africa, adult medical/surgical wards	CT-MEWS	142 vs 150	Appropriate response to deterioration; SAEs	Improved vital-sign documentation; no reduction in SAEs.
Umar et al. 2022 [11]	Nigeria, obstetric wards	Obstetric EWS	1200 vs 1200	Vital sign monitoring	Significant improvement in vital-sign monitoring in intervention hospital.
Agulnik et al. 2017 [12]	Guatemala, paediatric oncology	PEWS	157 vs 130	Clinical deterioration; PICU transfers	Reduced unplanned PICU transfers, deterioration events and PICU utilization.
Rosman et al. 2019 [13]	Rwanda, tertiary paediatric hospital	PEWS-RL	70 vs 68	Clinical deterioration	Shorter hospital stay; PEWS-RL > 3 accurately identified high-risk patients.
Sheikh et al. 2017 [14]	Pakistan, obstetric centre	NEWS	100 vs 100	Maternal and neonatal outcomes	No significant differences in major maternal or neonatal outcomes.

### Results of individual studies

#### Heterogeneity

Due to the limited amount of included studies and the heterogeneity among the studies, a meta-analysis could not be performed. Instead, a narrative analysis was performed to summarize and interpret the findings. The synthesis was structured around the main outcomes of interest and presented in a thematic and descriptive manner.

#### Mortality

All five studies [10–14] investigated the effect of an EWS on mortality. However, none of these studies found statistically significant differences in mortality between the control arm and the intervention arm.

#### Length of hospital stay

Three studies [10,11,13] investigated the effect of EWS on the length of hospital stay. Two studies [10,13] showed that the length of the hospital stay was shorter in the intervention arm compared to the control arm. The first study [10] showed shorter hospital stays in the MEWS study group compared to the control group (average of 6.0 (±3.8) days vs 8.0 (±5.9) days). However, it was not mentioned whether this result was statistically significant. Rosman et al., stated that the median length of hospital stay in days (median IQR) was significantly shorter among case patients (15 (5–27)) than among controls (23 (17–32)), *p*-value of <0.01 [13].

One study [11] found no statistically significant difference in the mean length of the hospital stay between the intervention arm and the control arm 3.6 (±3.2) vs 2.4 (±1.8) before EWS implementation 3.7 (±3.5) vs 2.3 (±1.6) after EWS implementation.

#### Serious adverse events (SAEs)

One study [10] reported the number of SAEs before and after the implementation of an EWS. There were no significant differences between the intervention arm (*n*  =  5; 3.3%) and the control arm (*n*  =  3; 2.1%), with a *p*-value of 0.72 (Table 1).

#### (P)ICU admissions/transfers

There were three studies [10,12,14] that investigated the effectiveness of an EWS on (P)ICU admissions/transfers.

One study [12] found a statistically significant reduction of unplanned PICU admissions before and after PEWS implementation (157 before PEWS implementation vs 130 after PEWS implementation; 7.6% vs 5.7% of hospital admissions; *p*-value of 0.012) (Table 1). The mean PICU length of stay in was not significantly different between the two groups (8.8 vs 8.4  days, *p*  =  0.86)

Two studies [10,14] found no statistical differences in (P)ICU admissions/transfers after EWS implementation. One study [10] found that 1 of the 150 patients (0.7%) in the intervention arm was admitted to the ICU. Out of the 142 patients, no patients in the control group were admitted to the ICU. Due to the small numbers, nothing can be concluded about the significance of these numbers. Another study [14] stated that after the implementation of NEWS, there were no patients who needed admission to ICU or tertiary care. NEWS implementation there was 1 in 100 patients who needed transfer to a high level of care (*p*-value  >  0.99). Also, there were no babies who needed transfer to a high level of care before the implementation of NEWS, while after the implementation of NEWS there were 2 in 100 babies who needed transfer to a high level of care, with a *p*-value of 0.49 (Table 1).

Overall, no consistent improvement in mortality, SAEs, or ICU admissions were observed across studies following EWS implementation.

### Risk of bias and certainty of evidence

Based on the GRADE tool, there were two studies [13,14] with a low level of certainty. There were three studies [10–12] with a moderate level of certainty.

Risk of bias was assessed qualitatively for each included study. Most studies were observational or quasi-experimental in design, with inherent risks of selection bias, confounding, and lack of blinding. Sample sizes were generally small, and several studies used pre-post designs without concurrent control groups. Overall, the methodological quality of the included studies was considered low to moderate.

## Discussion

This systematic review evaluated the effectiveness of EWS in LMICs. Ultimately, only five studies met the inclusion criteria, reflecting the limited published evidence in these settings. This review highlights a critical gap between early recognition of patient deterioration and the capacity to respond effectively within LMIC health-care systems. Our findings suggest that EWS should not be considered a standalone intervention, but rather a component of a broader health system that requires adequate staffing, training, and escalation capacity. All five studies examined mortality as an outcome. However, none demonstrated a statistically significant reduction in mortality following EWS implementation. Similarly, no significant reduction in SAE was observed. These findings underscore that the effectiveness of EWS in LMICs depends primarily on the strength of the surrounding health-care system rather than the predictive performance of the scoring system itself. This is in line with the consensus reached among global experts about ‘Essential Emergency and Critical Care’. In which training and routine have an important role but cannot be seen separately from equipment, drugs, human resource, guidelines, infrastructure, etc. [15].

There are fundamental differences in health system capacity between high-income countries and LMICs. First, in high-income countries, EWS are typically embedded within well-established rapid response systems, supported by adequate staffing, structured escalation pathways, and immediate access to critical care resources [16,17]. In contrast, many LMIC health-care systems have limited availability of trained personnel, restricted ICU capacity, or lack even basic monitoring equipment [18,19]. In such contexts, early identification of deterioration does not translate into effective intervention. Thus, while EWS may improve recognition of deteriorating patients, system and health-care limitations might prevent better outcome. Second, to have an effective EWS system, structured training programs, and continuous audit and feedback mechanisms are required. In LMICs, implementation may rely more heavily on paper-based systems, variable training, and inconsistent adherence to escalation protocols due to lack of continuous education.

Despite the absence of mortality benefit, some secondary outcomes were promising. Two studies demonstrated significant reductions in hospital stay, while one study showed a significant decrease in (P)ICU admissions/transfers. These findings align with earlier studies demonstrating the discriminative ability of EWS in acute care settings in LMICs [20]. Although this resulted not in improved survival rates, it suggests that EWS may contribute to more efficient patient triage and resource utilisation.

Importantly, qualitative findings indicated high levels of medical health-care providers with EWS implementation [11,14]. Similar observations have been reported in Guatemala, where nurses described feeling more empowered to assess patients, make decisions, and communicate concerns to their supervisors [21]. A study conducted in Peru, Mexico, El Salvador, and Ecuador concluded initial barriers such as, staff resistance to change, inadequate resources, and complexity of PEWS. However, all barriers were converted to enablers, and the overall perception of the PEWS in these centres was positive [22]. However, one of the included studies [10] reported no improvement in nurses’ ability to identify clinical deterioration. The overall perception of EWS in LMIC is positive. The scores and protocols empower nurses to talk to doctors more easily in an often hierarchic setting.

### Implementation challenges

Implementation of EWS in LMICS faces several barriers, including insufficient training, resource constraints, and chronic workforce shortages. For EWS to function effectively, training, education, and patient monitoring are essential. Furthermore, integrating EWS into existing health-care systems requires addressing infrastructure limitations and fostering collaboration among health-care teams.

Training-related factors have a crucial role in implementation success. The timing, duration, and intensity of training, as well as continuous audit and feedback mechanisms strongly influence the outcome. One included study reported no benefit of EWS implementation; however, effectiveness was assessed after a 2-hour training session. A comprehensive 8-hour training had been planned but could not be delivered due to staff shortage [10]. In contrast, two studies [11,22] showed positive results with a significant decrease in the vital sign error rate after a more comprehensive training, ongoing monitoring, and refresher courses. These findings suggest that inadequate training may lead to an underestimation of EWS effectiveness.

Workforce shortages represent another major challenge. In many LMIC hospitals, nurse-to-patient ratios on general wards may exceed 1:20, making frequent monitoring and documentation of vital signs difficult [23,24]. Two studies [10,11] addressed nursing shortage as a limiting factor to effective monitoring of vital signs and using the EWS. A study in Malawi [25] used Vital Sign Assistance (VSA’s) to measure vital signs and calculate the score. Where after training, the frequency of vital monitoring did not improve for the nurses. It increased 3.6-fold for the VSA’s. Taking vital signs is a narrow and repetitive task, therefore it is very suitable for task shifting to a VSA and relieving the pressure of the nursing staff.

### Limitations and biases

This review has several important limitations. First, the small number of included studies and the heterogeneity among them prevented a meta-analysis. Second, in some studies bias may have been introduced by methodological limitations. Small sample sizes, pre-post study designs without concurrent controls, observer bias, confounding variables, and selection bias are likely. Taken together, these limitations highlight the need for more rigorous, adequately powered studies that evaluate both clinical outcome and implementation process of EWS in LMIC. These limitations reflect the current state of available evidence rather than the shortcomings of the review methodology.

## Conclusion

Current evidence does not demonstrate improved patient outcomes following implementation of EWS in LMICs. However, this finding is based on a limited number of heterogeneous studies with varying methodological quality. The absence of observed benefits may be explained by contextual factors, including limited health-care resources, insufficient training, and restricted escalation capacity, which may prevent effective implementation of EWS. These findings suggest that EWS alone may be insufficient to improve patient outcomes without adequate health system support.

Future research should prioritise adequately powered multicentre studies. Equally, interesting would be an implementation-focused synthesis using also uncontrolled observational and implementation studies to focus not only on clinical outcomes but also factors such as training, monitoring, contextual factors, and response capacity.

## Supplementary Material

PRISMA_2020_checklist EWS SR.docx

Supplementary file 1.docx

## Data Availability

All data are available on request from the corresponding author.
