# Supplementary material for: Vital sign-based Early Warning Scores in low- and middle-income countries: a systematic review of clinical effectiveness and implementation challenges
Source: Glob Health Action. 2026 Jun 17;19(1):2690321. doi: 10.1080/16549716.2026.2690321 (PMC13276808; doi:10.1080/16549716.2026.2690321)
Supplement: Supplementary file 1.docx [file ZGHA_A_2690321_SM0954.docx]

**Supplementary Appendix 1**

**Pubmed search**

("Early Warning Score"[MeSH Terms] OR "Early Warning Score"[Title/Abstract] OR "EWS"[Title/Abstract] OR "MEWS"[Title/Abstract] OR "NEWS"[Title/Abstract] OR "risk stratification"[Title/Abstract] OR "risk assessment"[MeSH Terms]) AND ("Mortality"[MeSH Terms] OR "Mortality"[Title/Abstract] OR "serious adverse events"[Title/Abstract] OR "ICU admissions"[Title/Abstract] OR "Intensive Care Units"[MeSH Terms] OR "Length of Stay"[MeSH Terms] OR "hospitalization"[MeSH Terms] OR "treatment outcome"[MeSH Terms]) AND ("Developing Countries"[MeSH Terms] OR "low-income countries"[Title/Abstract] OR "middle-income countries"[Title/Abstract] OR "LMICs"[Title/Abstract] OR "resource-limited settings"[Title/Abstract] OR "underdeveloped countries"[Title/Abstract])

**EMBASE search**

('early warning score':ti,ab OR 'EWS':ti,ab OR 'MEWS':ti,ab OR 'NEWS':ti,ab OR 'risk stratification':ti,ab OR 'risk assessment'/exp) 
AND 
('mortality'/exp OR 'mortality':ti,ab OR 'serious adverse events':ti,ab OR 'ICU admission':ti,ab OR 'intensive care unit'/exp OR 'length of stay'/exp OR 'hospitalization'/exp OR 'treatment outcome'/exp)
AND 
('developing countries'/exp OR 'low-income countries':ti,ab OR 'middle-income countries':ti,ab OR 'LMICs':ti,ab OR 'resource-limited settings':ti,ab OR 'underdeveloped countries':ti,ab)

**The Cochrane Library search**

("early warning score" OR "EWS" OR "MEWS" OR "NEWS" OR "risk stratification" OR "risk assessment") 
AND 
("mortality" OR "serious adverse events" OR "ICU admissions" OR "intensive care unit" OR "length of stay" OR "hospitalization" OR "treatment outcome") 
AND 
("developing countries" OR "low-income countries" OR "middle-income countries" OR "LMICs" OR "resource-limited settings" OR "underdeveloped countries")
